# Supplementary material for: The Vertical Metabolic Activity and Community Structure of Prokaryotes along Different Water Depths in the Kermadec and Diamantina Trenches
Source: Microorganisms. 2024 Mar 30;12(4):708. doi: 10.3390/microorganisms12040708 (PMC11052081; doi:10.3390/microorganisms12040708)
Supplement: Supplementary file 1 [file microorganisms-12-00708-s001.zip › microorganisms-2870315-supplementary.pdf]

Supplementary Material

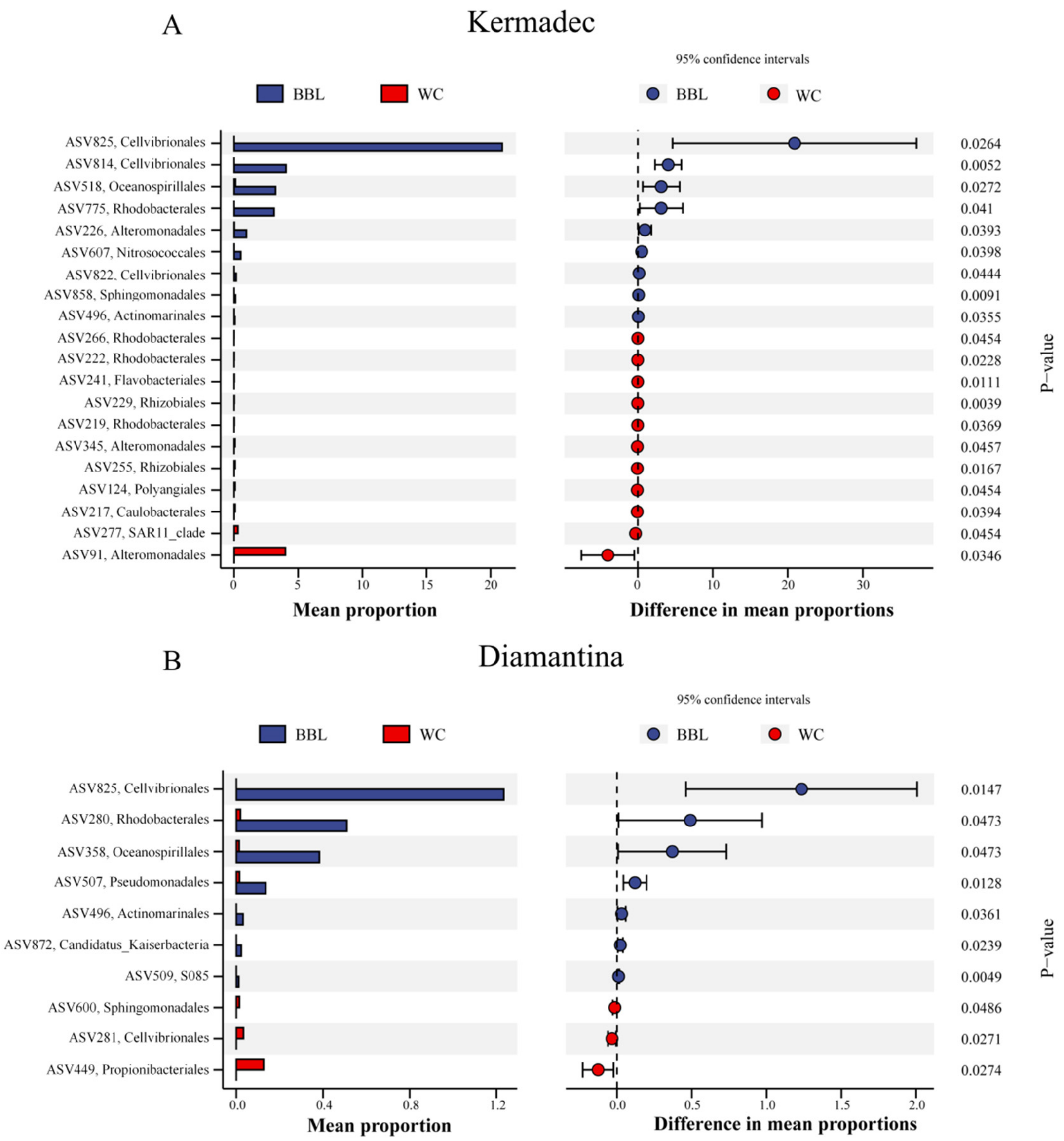

Figure S1: Extended error plot between the WC and BBL for the Kermadec Trench (A) and Diamantina Trench (B) visualized through STAMP software. Mean proportions in different categories were displayed in the left bar graph. The colored circles (brown and blue) showed the 95% confidence intervals calculated using the Welch's t-test.

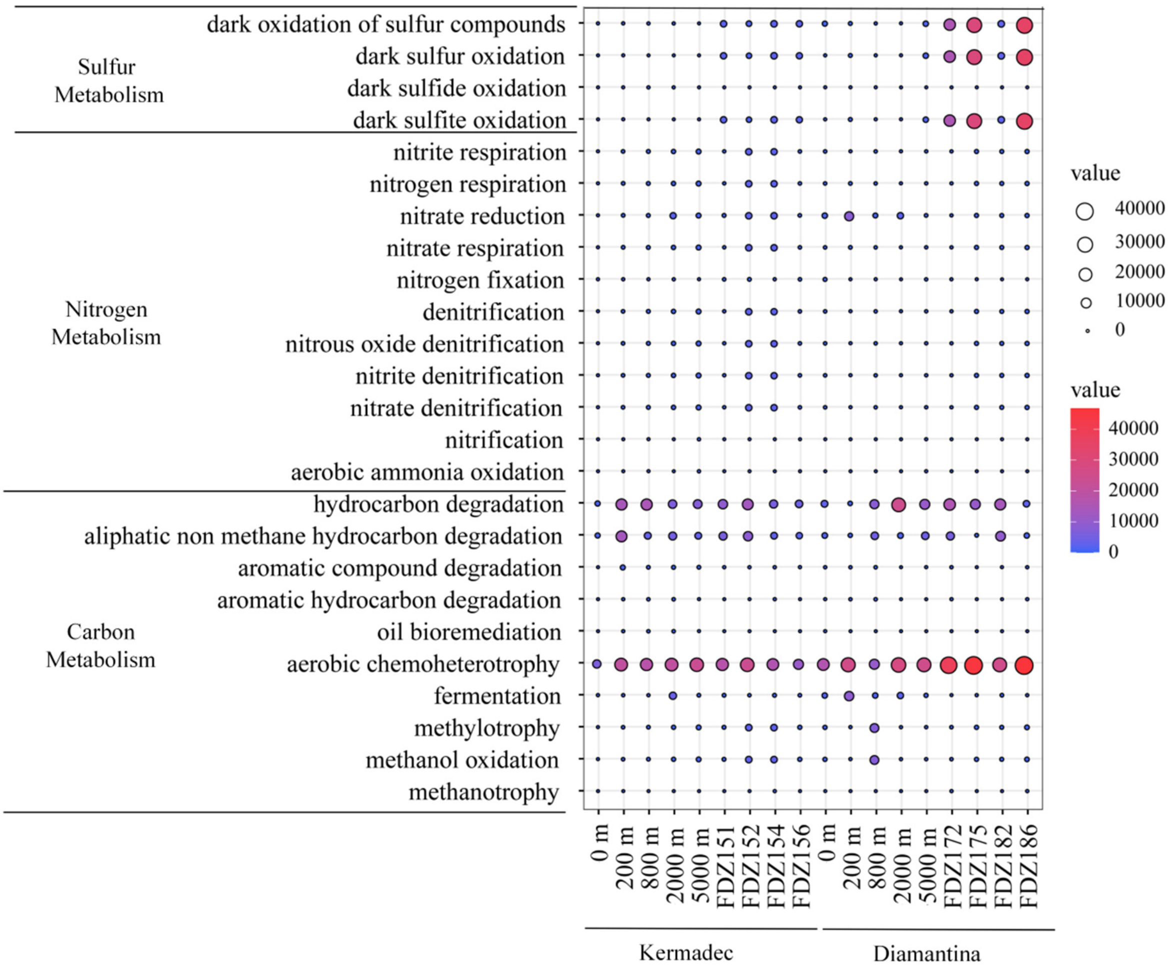

Figure S2: Summary of the mean abundance of pathways associated with sulfur, nitrogen, carbon metabolize in the WC and the BBL of the Kermadec Trench and the Diamantina Trench.
